# Fixation using alternative implants for the treatment of hip fractures (FAITH-2): design and rationale for a pilot multi-centre 2 × 2 factorial randomized controlled trial in young femoral neck fracture patients

**DOI:** 10.1186/s40814-019-0458-x

**Published:** 2019-05-28

**Authors:** FAITH-2 Investigators, Gerard P. Slobogean, Sheila Sprague, Sofia Bzovsky, Diane Heels-Ansdell, Lehana Thabane, Taryn Scott, Mohit Bhandari, Earl Bogoch, P. J. Devereaux, Gordon Guyatt, Martin J. Heetveld, Kyle Jeray, Susan Liew, Robert V. O’Toole, Andrew N. Pollak, Emil H. Schemitsch, Marc Swiontkowski, Dale Williams, Kim Madden, Alisha Garibaldi, Lisa Buckingham, Nicole Simunovic, Martí Bernaus, Eleanor M. Pullenayegum, Matthew Menon, David Teague, Gregory J. Della Rocca, Richard J. Jenkinson, Hans J. Kreder, David J. G. Stephen, Markku Nousiainen, Diane Nam, Patrick Henry, John Iazzetta, Katrina Hatzifilalithis, Katrine Milner, Monica Kunz, Aimee Theriault, Wesley Ghent, Araby Sivananthan, Fathima Adamsahib, Danyella Dias, Abeer Wasim, Ravianne Tuazon, Katerina Polihronidis, Aaron Nauth, Michael D. McKee, Jeremy A. Hall, Daniel Whelan, Timothy Daniels, Sarah Ward, Henry Ahn, James Waddell, David Walmsley, Amit Atrey, Laura Parsons, Ann Dowbenka, Gitana Ramonas, Milena R. Vicente, Jennifer T. Hidy, Paril Suthar, Melanie MacNevin, Darius Viskontas, Robert McCormack, Farhad Moola, Bertrand Perey, Trevor Stone, Kelly Apostle, Dory Boyer, H. Michael Lemke, Mauri Zomar, Karyn Moon, Raely Pritchard, Brenda Chen Fan, Bindu Mohan, Pierre Guy, Kelly Lefaivre, Peter O’Brien, Henry Broekhuyse, Piotr Blachut, Dean Malish, Jeffrey Potter, Raman Johal, Irene Leung, Benita Okocha, Jessica Peattie, Abdullah Mamun, Steven Papp, Wade Gofton, Allan Liew, Sherry Weir, Darren M. Roffey, Nicole Harris, Julia Foxall, Andrew Furey, Keegan Au, Daniel Squire, Peter Rockwood, Ashley Buck, Karen Ryan, Garrett Wells, Zeta Hannaford, Valisha Keough, Erin Baker, Sarah Anthony, Mark Richardson, Thomas DiPasquale, Paul Muccino, Troy Caron, Adrienne Brandon, Krystal Swasey, Tara Moore, David Hubbard, John France, Michelle A. Bramer, Brock Lindsey, Benjamin Frye, Matthew Dietz, Adam Klein, George K. Bal, E. Barry McDonough, John P. Lubicky, Scott Daffner, T. Ryan Murphy, Lisa Giblin Sutton, Sheila Rye, Nina Clovis, Sherri Davis, Robert A. Hymes, Cary C. Schwartzbach, A. Stephen Malekzadeh, Jeff E. Schulman, Michael Holzman, Anastasia Lialios-Ramfos, Lolita Ramsey, Sharon Haaser, Gudrun Mirick Mueller, David Templeman, Andrew Schmidt, Tzivia Leviton, Jerald R. Westberg, Saam Morshed, Eric Meinberg, Paul Toogood, David Shearer, Murat Pekmezci, Paul Knaus, Sara McFarland, Tigist Belaye, Eleni Berhaneselase, Johnathan Kwong, Christina Boulton, Christopher LeBrun, Jason W. Nascone, Marcus F. Sciadini, Raymond Pensy, Theodore Manson, W. Andrew Eglseder, Andrea L. Howe, Daniel Connelly, Katherine Ordonio, Raza Zaidi, Yasmin Degani, Merryjessica Fuerst, Carrie Schoonover, George Reahl, Peter Berger, Ryan Montalvo, Dimitrius Marinos, Daniel Mascarenhas, Joshua Rudnicki, Clifford B. Jones, Sarim Ahmed, Norman Chutkan, Michael Lucero, Jason Lowe, Russell Meldrum, Niloofar Dehghan, Ryan DiGiovanni, Pierce Johnson, Sean Karr, Sean Mitchell, Robert Walker, Debra L. Sietsema, Cyndi Ventry, Susan Mauro, Karen Walsh, Bonnie Sumner, Taylor Dykes, Michael Duran, Blake Eyberg, Joseph Walker, Collin Barber, Jessica Burns, Jill Goodwin, Ryan Shelhamer, Joshua Hustedt, Damien Richardson, Julie Robbins, Greg E. Gaski, Todd O. McKinley, Walter W. Virkus, Laurence B. Kempton, Anthony T. Sorkin, Roman Natoli, Charles Lieder, Landon Fine, Lauren C. Hill, Krista M. Brown, Lakye Deeter, Jennifer Hagen, André Spiguel, Kalia Sadasivan, Matthew Patrick, Melissa Johnson, Susan Beltz, Elizabeth Agustin, Chris Koenig, Sonya Brisbane, Tudor V. Tufescu, Nigar Sultana, Ryan Bicknell, Heather Grant, Fiona Howells, Todd M. Oliver, Michelle Vogt, Vicki Jones, Tina Carter, Edward Westrick, Traci Salopek, Aaron Perdue, Joel J. Gagnier, James Goulet, Mark Hake, Jaimee Gauthier, Christopher Robbins, Jianyu Lai, Anne Mak, Zoe Murdoch, Gerard P. Slobogean, Sheila Sprague, Sofia Bzovsky, Diane Heels-Ansdell, Lehana Thabane, Taryn Scott, Mohit Bhandari

**Affiliations:** 1 R Adams Cowley Shock Trauma Center, Department of Orthopaedics, University of Maryland School of Medicine, 22 South Greene Street, Baltimore, MD 21201 USA; 2 Division of Orthopaedic Surgery, Department of Surgery, McMaster University, 1280 Main Street West, Hamilton, ON L8S 4L8 Canada; 3 Department of Health Research Methods, Evidence, and Impact, McMaster University, 1280 Main Street West, Hamilton, ON L8S 4L8 Canada

**Keywords:** Clinical protocols, Femoral neck fractures, Fracture fixation, internal, Vitamin D, Randomized controlled trial

## Abstract

**Background:**

Femoral neck fractures in patients ≤ 60 years of age are often very different injuries compared to low-energy, hip fractures in elderly patients and are difficult to manage because of inherent problems associated with high-energy trauma mechanisms and increased functional demands for recovery. Internal fixation, with multiple cancellous screws or a sliding hip screw (SHS), is the most common treatment for this injury in young patients. However, there is no clinical consensus regarding which surgical technique is optimal. Additionally, there is compelling rationale to use vitamin D supplementation to nutritionally optimize bone healing in young patients. This pilot trial will determine feasibility and provide preliminary clinical data for a larger definitive trial.

**Methods:**

We will conduct a multicenter, concealed randomized controlled pilot study, using a 2 × 2 factorial design in 60 patients aged 18–60 years with a femoral neck fracture. Eligible patients will be randomized in equal proportions to one of four groups: 1) SHS and vitamin D supplementation (4000 international units (IU) daily dose) for 6 months, 2) cancellous screws and vitamin D supplementation (4000 IU daily dose) for 6 months, 3) SHS and placebo, and 4) cancellous screws and placebo. Participants will be followed for 12 months post-fracture. Feasibility outcomes include initiation of clinical sites, recruitment, follow-up, data quality, and protocol adherence. Clinical outcomes, for both the pilot and planned definitive trials, include a composite of patient-important outcomes (re-operation, femoral head osteonecrosis, severe femoral neck malunion, and nonunion), health-related quality of life and patient-reported function, fracture healing complications, and radiographic fracture healing. A priori success criteria have been established. If the pilot study is deemed successful, study participants will be included in the definitive trial and clinical outcomes for the pilot will not be analyzed. If the pilot study is not deemed successful, clinical outcome data will be analyzed.

**Discussion:**

Results of this study will inform the feasibility of a definitive trial. If clinical outcome data are analyzed, they will be disseminated through a publication and presentations.

**Trial registration:**

The FAITH-2 trial, described as a definitive trial, was registered at ClinicalTrials.gov (NCT01908751) prior to enrollment of the first participant.

**Electronic supplementary material:**

The online version of this article (10.1186/s40814-019-0458-x) contains supplementary material, which is available to authorized users.

## Background

### Femoral neck fractures in young patients

Femoral neck fractures in patients 60 years of age or younger have a different presentation compared to low-energy, elderly hip fractures. Femoral neck fractures in young patients are difficult injuries to manage because of the inherent problems associated with their typical high-energy trauma mechanism and increased patient functional demands for recovery. Compared to elderly patients with femoral neck fractures, younger patients often present with a displaced and vertically orientated fracture. These fracture characteristics make reduction more challenging and internal fixation more susceptible to failure. Although hip arthroplasty has become a successful treatment for elderly fractures, joint replacement has not been considered an optimal treatment option for younger patients due to concerns of implant longevity and higher failure rates in active patients less than 55 years old [1].

### Complications with femoral neck fractures in young patients

Despite deliberate efforts to preserve the native hip by performing internal fixation for nearly all femoral neck fractures in young patients (ages < 60 years), fracture healing complications are unacceptably high. In a recent meta-analysis of 42 studies pooling the results of internal fixation for young femoral neck fractures, an 18% incidence of re-operation was reported. [2] Femoral head avascular necrosis (AVN) occurred in 14% of cases, and nonunion occurred in 9% of cases. In addition, even patients who do not experience a fracture healing complication may still experience poor functional outcomes. This occurs because approximately 30% of patients heal with more than 10 mm of fracture shortening, and shortening greater than 5 mm has been associated with clinically significant decreases in functional outcome. [3, 4] These findings have been confirmed by a recently completed prospective multi-center cohort study of 142 femoral neck fracture patients aged 18–55 in China [5]. In this population, it was found that severe femoral neck shortening (≥ 10 mm) occurred in 13% of patients and was associated with worse functional outcome scores [5]. Therefore, the young femoral neck fracture population is at great risk for experiencing significant fracture healing complications, re-operations, and lifelong morbidity.

### The role of vitamin D in acute fracture healing

Although the fracture pattern and displacement characteristics of femoral neck fractures in young patients have traditionally been viewed as the main determinants of healing complications, emerging literature suggests many trauma patients may already be at significant nutritional risk for fracture healing difficulties. Recent literature suggests that up to 80% of non-elderly fracture patients are vitamin D insufficient [6–8]. This is a potentially important risk factor for complications since vitamin D is essential to musculoskeletal health. Experimental animal studies suggest vitamin D has a role in the fracture callus formation, mechanical bone strength, and time to union [9–11]. Clinical studies have also suggested that vitamin D supplementation may increase the callus volume of proximal humerus fractures [12], increase the number and diameter of type II muscle fibers [13], and can improve wound healing [14]. The high prevalence of vitamin D insufficiency among young trauma patients combined with the critical microvascular injury at the femoral neck suggests that these fracture patients may face significant barriers to healing and achieving good functional outcomes.

### Controversies in treatment of young femoral neck fractures

Although internal fixation is the standard of care for the majority of femoral neck fractures in young patients, significant controversy exists when determining the optimum method of fixation. The most commonly used internal fixation devices are multiple cancellous screws or a sliding hip screw (SHS). To our knowledge, there are two small, randomized controlled trials (RCT) comparing multiple screws to a SHS in young femoral neck fractures. These studies are hindered by very small sample sizes, methodology limitations, and data that was published more than 25 years ago; however, they independently suggest better outcomes in the multiple screw group [15, 16]. In contrast to the findings of these RCTs, more recent uncontrolled retrospective studies have suggested substantially better results with SHS fixation [17–20]. A recent attempt to obtain expert consensus and resolve the treatment debate among surgeons further highlighted the current controversies. In a survey of more than 500 orthopedic surgeons, an equal preference for the two implants was reported during the treatment of displaced fractures [21]. This clinical equipoise mirrors the controversy in the published literature.

A similar lack of consensus and research data to guide the management of vitamin D insufficiency in young fracture patients exists. According to several observational studies, up to 75% of healthy adult fracture patients between the ages of 18 and 50 have insufficient or deficient serum 25-hydroxyvitamin D [25(OH)D] levels (< 30 ng/mL) and that vitamin D levels may continue to decrease after sustaining a fracture [9, 22–26]. It has been hypothesized that vitamin D supplementation taken after a fracture may improve fracture healing outcomes [12, 13]. To date, there only exists a small pilot RCT suggesting that a single loading dose of vitamin D may diminish the risk of nonunion [27–33]. Although the biologic rationale to use vitamin D supplementation to nutritionally optimize the bone health of young femoral neck fracture patients is compelling, more clinical research is needed [34]. Since the optimal dose and frequency is currently unknown, 4000 international units (IU) daily was selected for our pilot study because it corresponds to the highest tolerable daily dose identified by the Institutes of Medicine [35].

### Summary of rationale for RCT

Femoral neck fractures in younger patients (age < 60) are challenging injuries to treat. There is a high incidence of fracture healing complications in this population: 18% re-operation, 14% femoral head AVN, 9% nonunion, and 30% severe fracture shortening [2]. The majority of these fractures are treated with internal fixation using cancellous screws or a SHS. Currently, there is no consensus among surgeons as to preferred treatment, and the literature remains conflicted regarding the optimum fixation implant. Furthermore, there is emerging evidence that the majority of young trauma patients are vitamin D insufficient and there is a potential role for optimization of vitamin D levels during fracture healing. The burden of femoral neck fractures in young patients remains unacceptably large due to high complication rates and poor functional outcomes. Conflicting clinical data, a lack of surgeon consensus, and a need for novel strategies to reduce young adult hip fracture morbidity support a definitive RCT.

### Rationale for a pilot study

As femoral neck fractures in young patients are a rare injury, multi-national collaboration will be necessary to meet the projected sample size for the definitive trial. Currently, there is no consensus among surgeons, or in the literature, as to the optimal implant for fixation. Furthermore, there is emerging evidence that young trauma patients are vitamin D insufficient and that there is a role of vitamin D supplementation during fracture healing. Conflicting clinical data, a lack of surgeon consensus, and a need for novel strategies to reduce young adult hip fracture morbidity support the undertaking of a definitive RCT. Additionally, there is limited data on dosing levels, compliance with daily dosing, and whether daily vitamin D dosing is sufficient to improve fracture healing outcomes in the young trauma population.

Therefore, our rationale for conducting a pilot study is to determine feasibility of a definitive RCT. Given the high cost of conducting a definitive trial, it is necessary to address this question before initiating a larger trial.

## Study design

### Overview of study design

We propose a pilot study to determine the feasibility of a definitive 2 × 2 factorial design RCT comparing two alternative surgical techniques and vitamin D supplementation versus placebo for the treatment of femoral neck fractures in young adult patients (ages 18–60). Participants will be randomized to one of two surgical treatment arms: multiple cancellous screws (cancellous screw group) or a single large diameter screw and side plate (SHS group). Furthermore, participants will be randomized to receive a nutritional supplement (vitamin D supplementation) or placebo. The sample size for the pilot study is 60 patients. Feasibility outcomes will include the initiation of clinical sites, rate of participant enrollment (enrollment of 60 patients within 12 months), proportion of participants with complete follow-up at 12 months post-fracture, level of data quality, and rate of protocol adherence. Participants will be followed for 12-months post-fracture. Clinical outcomes include a composite of patient-important outcomes (re-operation, femoral head osteonecrosis, severe femoral neck malunion, and nonunion), health-related quality of life (HRQL) and patient-reported function, fracture healing complications, and radiographic fracture healing (Table 1). The Fixation using Alternative Implants for the Treatment of Hip Fractures (FAITH-2) trial, described as a definitive trial, is registered at ClinicalTrials.gov (Identifier NCT01908751) and has received ethics approval from the Hamilton Integrated Research Ethics Board (HIREB# 13–807) and each participating clinical sites’ local Research Ethics Board (REB) or Institutional Review Board (IRB). This protocol paper adheres to the SPIRIT checklist (Additional file 1) as a guide for reporting.

**Table 1:** Schedule of events (SPIRIT)

Assessment	Screening	Enrollment (baseline)	Surgery	Post-operative	Week 6	Month 3	Month 6	Month 9	Month 12
Informed consent	X								
Medical history	X								
Anterior-posterior and lateral X-rays of proximal femur	X			X	X	X	X	X	X
Physical Exam/injury assessment	X								
Screening form	X								
Randomization form		X							
Pre-operative form		X							
Surgery (SHS or cancellous screws)			X						
Surgical forms			X						
Hospital discharge form				X					
Vitamin D or placebo supplementation^b^				X	X	X	X		
Follow-up visit forms				X	X	X	X	X	X
Assessment for re-operations				X	X	X	X	X	X
Assessment of fracture healing complications					X	X	X	X	X
Assessment of fracture healing					X	X	X	X	X
Hip Outcome Score (HOS)				X^a^	X	X	X	X	X
Short-Form 12 (SF-12)				X^a^	X	X	X	X	X
Radiographic Union Score for Hip (RUSH)							X	X	X
Assessment of fracture healing of the ipsilateral femoral shaft fracture^c^					X	X	X	X	X
Assessment for fracture-related adverse events			X	X	X	X	X	X	X
Assessment for serious adverse events			X	X	X	X	X	X	X
Assessment for planned re-operations									X

^a^Asks about participant’s function prior to their hip fracture

^b^Nutritional supplementation will be administered upon hospital discharge or within 2 weeks of the participant’s surgery, whichever comes first. Ideally, participants should be administered the nutritional supplementation as soon as possible following their surgery to repair their femoral neck fracture

^c^For participants with an ipsilateral femoral shaft fracture

### Primary research question for the pilot study

Is it feasible to conduct a definitive RCT that evaluates whether fixation with a SHS and nutritional supplementation with vitamin D independently lower the risk of patient important outcomes (re-operation, femoral head osteonecrosis, severe femoral neck malunion, and nonunion) during the 12-month post-injury follow-up period in young adults (ages 18–60) with femoral neck fractures?

### Primary clinical research questions for the pilot study

1. In patients with femoral neck fractures, will the risk of patient important outcomes, defined as a composite of re-operation, femoral head osteonecrosis, severe femoral neck malunion, and nonunion, be lower in the SHS treatment arm compared to the cancellous screw treatment arm 12-months post-injury?
2. In patients with femoral neck fractures, will the risk of patient important outcomes, defined as a composite of re-operation, femoral head osteonecrosis, severe femoral neck malunion, and nonunion, be lower in the vitamin D treatment arm compared to the placebo arm 12-month post-injury?

### Feasibility objectives for the pilot study

Feasibility objectives for the pilot study will be to assess the initiation of clinical sites, rate of participant enrolment, proportion of participants with complete follow-up at 12 months post-fracture, level of data quality, and rate of protocol adherence (e.g., the number of errors in randomization, the number of crossovers between SHS and cancellous screw treatment groups, and adherence to the daily vitamin D supplementation).

## Methods

### Study setting

This pilot study will be conducted at approximately 25 academic and community hospitals across North America, Australia, Europe, and Asia that treat femoral neck fractures in young adults.

### Inclusion criteria

Patients who meet all the following criteria will be included in the study:

1. Adult men or women ages 18 to 60 years.
2. Fracture of the femoral neck.
3. Fracture amenable to both surgical treatments (SHS and cancellous screws).
4. Operative treatment within 7 days of injury.
5. Provision of informed consent by patient or substitute decision maker.

### Exclusion criteria

Patients who meet any one or more of the following criteria will be excluded from the study:

1. Patients with previously diagnosed osteoporosis.
2. Fracture-dislocation of the femoral neck and hip joint.
3. Planned antegrade nailing of an ipsilateral femoral shaft fracture (if present).
4. Current infection around the hip (i.e., soft tissue or bone).
5. Stress fracture of the femoral neck.
6. Pathologic fractures secondary to neoplasm or other bone lesion.
7. Patients with known or likely undiagnosed disorders of bone metabolism such as Paget’s disease, osteomalacia, osteopetrosis, osteogenesis imperfecta, etc.
8. Patients with hyperhomocysteinemia.
9. Patients with an allergy to vitamin D or another contraindication to being prescribed vitamin D.
10. Patient is currently taking an over the counter drug and/or food supplement that contains vitamin D and is unable or unwilling to discontinue its use for this study.
11. Likely problems, in the judgment of the attending surgeon, with maintaining follow up (e.g., patients with no fixed address, plans to move out of town). This may include patients with severe mental disorders and drug addictions without adequate support.
12. Pregnancy.
13. Patient is incarcerated.
14. Patient is not expected to survive injuries.
15. The attending surgeon believes the patient should be excluded because they are involved in a conflicting clinical trial.

We will include all femoral neck fracture patterns (subcapital, midcervical, or basicervical). Ipsilateral femoral shaft fractures treated with retrograde nailing or plating will be eligible for inclusion. Patients with multiple trauma will also be eligible for inclusion. Patients with bilateral femoral neck fractures will be eligible for inclusion; however, only the most severe eligible fracture will be included (defined as the most displaced fracture, as determined by the attending surgeon).

### Recruitment and screening

All patients presenting to participating surgeons between the ages of 18 to 60 years with a femoral neck fracture will be screened. Potentially eligible patients will be approached to participate in the pilot FAITH-2 trial. All screened patients will be classified as 1) excluded (if they subsequently do not meet the eligibility criteria), 2) missed (eligible but not randomized due to error), 3) or included (eligible and randomized). The study coordinator or designee will obtain informed consent for participation in the study using local IRB/REB approved informed consent forms.

### Allocation of patients to study groups

Eligible patients will be randomized in equal proportions to one of four treatment groups: 1) cancellous screws with vitamin D_3_ supplementation for 6 months, 2) cancellous screws with placebo supplementation for 6 months, 3) SHS with vitamin D_3_ supplementation for 6 months, or 4) SHS with placebo supplementation for 6 months. Allocation will be concealed using a centralized 24-h computerized randomization system that will allow Internet-based allocation. The treatment allocation will be stratified on the following prognostic factors to ensure balance between the intervention groups: 1) undisplaced or displaced femoral neck fractures, 2) presence or absence of an ipsilateral femoral shaft fracture, and 3) geographic region of recruiting center (industrializing countries versus industrialized countries).

### Surgical interventions

#### Multiple cancellous screws

**Fig. 1:**
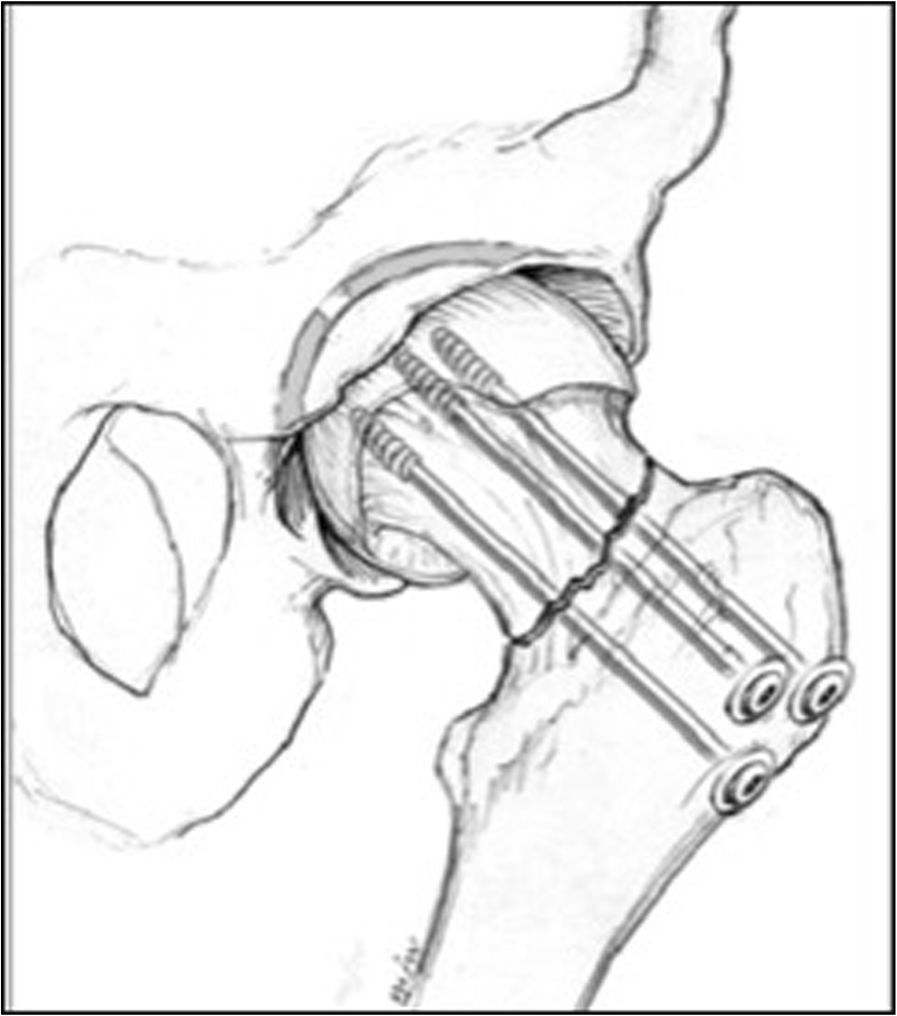
Multiple cancellous screws

Participants allocated to the cancellous screw group will receive multiple threaded screws (with a minimum of three screws and a minimum diameter of 6.5 mm) (Fig. 1). Any threaded screw or hook pin as well as buttress plates will be permitted. The number of screws, screw configuration, reduction technique, implant manufacturer, use of buttress plates, decision to perform a capsulotomy, use of injectable bone substitutes, use of bone grafts, or aspiration of an intracapsular hematoma will be documented but not prescribed due to lack of evidence favoring any of these approaches.

#### Sliding hip screws

**Fig. 2:**
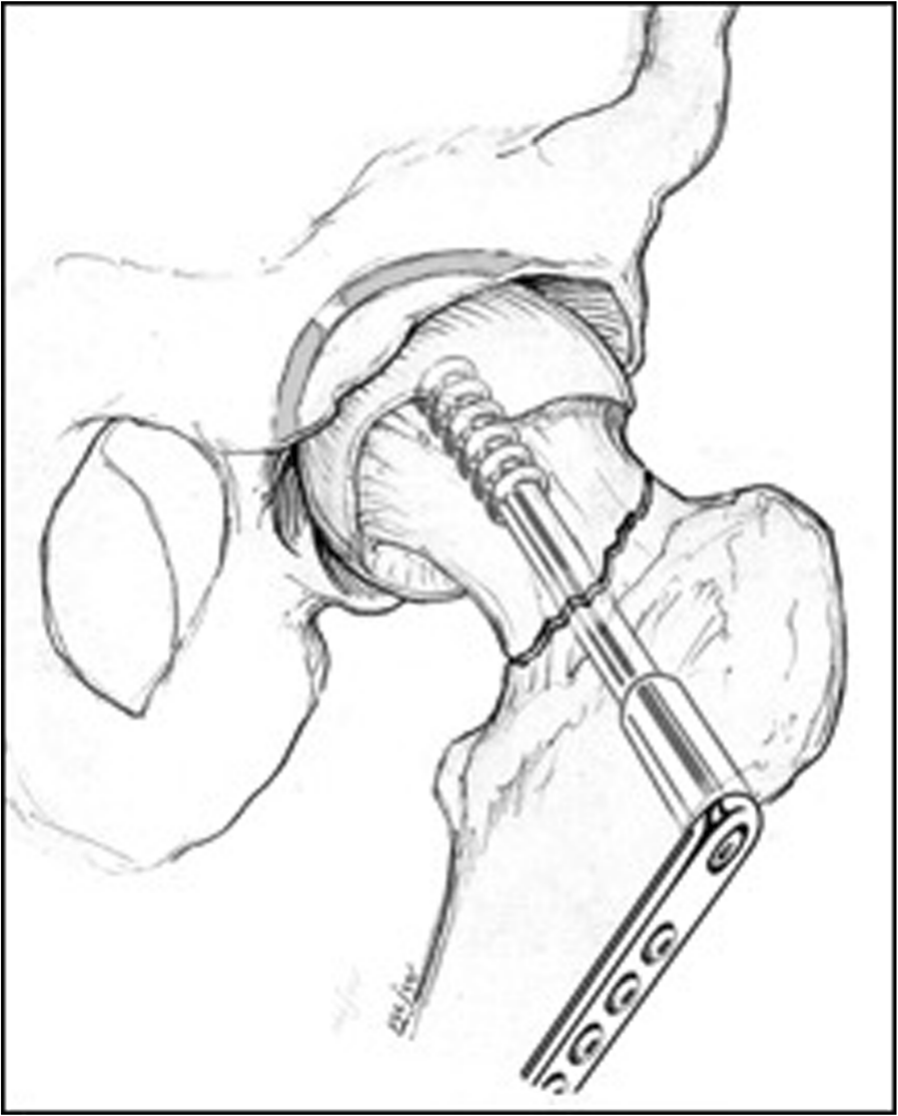
Sliding hip screw

Participants allocated to the sliding hip screw (SHS) group will receive a single larger diameter partially threaded screw affixed to the proximal femur with a sideplate using a minimum of two screws for fixation (Fig. 2). Surgeons will be permitted to use any fixed-angle plate construct which includes a large diameter screw or blade that can slide within the plate. Surgeons will be allowed to use derotational screws and buttress plates. The use of a compression screw, implant manufacturer, reduction technique, decision to perform a capsulotomy, use of injectable bone substitutes, use of bone grafts, and aspiration of intracapsular hematoma will be documented but not prescribed.

### Adherence to surgical intervention

Crossover rates between the SHS and multiple cancellous screw groups are likely to be low because both implants are inserted with similar techniques, and surgeon expertise in both techniques is likely to be very similar. Participants will be randomized as close to surgery as possible. Any participants who crossover will be analyzed in the group to which they were allocated, maintaining the intention to treat approach for the analysis.

### Nutritional supplementation

Nutritional supplementation will be administered upon hospital discharge or within 2 weeks of the participant’s femoral neck surgery, whichever comes first. Ideally, participants should be administered the nutritional supplementation as soon as possible following their surgery. Each participant will be provided with a 6-month supply of vitamin D_3_ supplementation or placebo.

Participants allocated to the vitamin D Group will receive a bottle of 2000 IU vitamin D_3_ drops (Ddrops®, Ddrops Company). Participants will be instructed to take two drops daily for 6 months, for a total daily dose of 4000 IU. All vitamin D_3_ supplement bottles will be labeled in a blinded manner according to Health Canada guidelines and Good Manufacturing Practice.

Participants in the placebo group will receive an identical bottle of placebo drops with no active ingredient. Similarly, they will be instructed to take two drops daily for 6 months. The placebo supplement is also manufactured by the Ddrops Company. All placebo supplement bottles will be labeled in a blinded manner according to Health Canada guidelines and Good Manufacturing Practice.

Crossovers are unlikely between the nutritional supplementation groups as participants and surgeons will be blinded to the vitamin D_3_ and placebo treatments. Additionally, the importance of treatment compliance will be discussed with participants at each follow-up visit. Participating health care providers and study personnel will be instructed that prescribing study participants additional vitamin D is prohibited. Participants will also be instructed not to take additional supplements containing vitamin D for the duration of the trial. Previous research has demonstrated a 96% adherence to daily vitamin D self-administration in adults [36]. Additionally, the nutritional treatment arm represents a pragmatic effectiveness comparison, and given the placebo-controlled blinding, there is no reason to suspect differential compliance between the treatment groups will occur.

### Outcome measures

#### Feasibility outcome measures

Feasibility outcomes for the pilot study include the initiation of clinical sites, rate of participant enrolment, proportion of participants with complete follow-up at 12 months post-fracture, level of data quality, and rate of protocol adherence (e.g., the number of errors in randomization, the number of crossovers between SHS and cancellous screw treatment groups, and adherence to the daily vitamin D supplementation).

##### Criteria for success of feasibility

We will consider our pilot study a success in regard to feasibility if the following criteria are met: 1) initiation of clinical sites in North America, Europe, Asia, and Australia; 2) 60 patients enrolled over a 12-month period; 3) at least 90% of participants achieve follow-up at 12 months for the proposed primary outcome of the definitive trial (patient important outcomes); 4) at least 90% case report form completeness with no outstanding queries at 12 months, 5) less than 5% errors in randomization across sites; 6) at least 90% adherence to surgical technique; and 7) less than 5% errors in the treatment allocation of vitamin D supplementation.

#### Primary clinical outcome

The primary clinical outcome for the pilot study will be a composite of patient important outcomes that occur within the 12 months post-surgery follow-up period. The composite will include 1) re-operation, defined as any unplanned surgery related to the treatment of the femoral neck fracture; 2) femoral head osteonecrosis as reported on any follow-up medical imaging study (i.e., radiographs, magnetic resonance imaging, or other advanced imaging study); 3) severe femoral neck malunion, defined as shortening of > 10 mm in any plane on follow-up X-rays; and 4) nonunion, defined as the failure of the fracture to progress toward healing defined as a Radiographic Union Score for Hip (RUSH) score below 18 at 6 months or greater post-injury [37]. A participant will be classified as having the primary clinical outcome if they experience one or more of the above patient important outcomes.

#### Secondary clinical outcomes

The secondary outcomes for the pilot study will include 1) HRQL and patient-reported function, 2) fracture healing complications, and 3) radiographic fracture healing. HRQL will be assessed using the Short Form-12 (SF-12) which measures self-reported quality of life through an 8-domain profile of functional health and well-being, physical and mental health summary measures, and a preference-based health utility index [38]. Patient-important function will be assessed with the Hip Outcome Score (HOS) which measures self-reported functional status through 28 items and two sub-scales that pertain to activities of daily living (ADLs) or higher level activities such as those necessary to participate in sports [39].

Fracture healing complications will include wound healing problems, infection (superficial and deep), hardware failure, hardware breakage, painful hardware, and peri-prosthetic fracture. For radiographic fracture healing, the date of healing will be determined by an adjudicator. The adjudicator will consider a fracture as healed when there is obliteration of the fracture line by newly formed bone along the cortices and within the trabecular bone on anterior-posterior and lateral radiographs.

In addition, for patients with ipsilateral femoral shaft fractures, we will document the method of shaft fracture fixation. We will also record and have the independent adjudicator adjudicate the following outcomes for the femoral shaft fracture: 1) the time to radiographic fracture healing of the ipsilateral femoral shaft, 2) re-operation, and 3) fracture-related complications.

### Participant follow-up

Feasibility of the pilot study will be assessed over 12 months post-fracture. Participants will be followed as per the protocol for the planned definitive trial, which is summarized below. Participant follow-up visits will occur at enrollment (baseline), post-operative (24-h to 14-day window), 6 weeks (2-week to 8-week window), 3 months (2- to 4-month window), 6 months (5- to 7-month window), 9 months (7- to 11-month window), and 12 months (11 months or greater window) post-surgery (Table 1).

Additionally, at all follow-up visits, the participant will complete the SF-12 and HOS questionnaires. At the post-operative follow-up visit, participants will answer all questionnaires based on their pre-injury status. Questionnaires for subsequent follow-up visits will be answered based on the participant’s current status at the time of follow-up (Table 1).

For participants who have an ipsilateral femoral shaft fracture at each follow-up visit, we will document 1) the time to radiographic fracture healing of the ipsilateral femoral shaft, 2) re-operation, and 3) fracture-related complications including compartment syndrome, wound healing problems, infection (superficial or deep), hardware failure, hardware breakage, malunion, nonunion, and prolonged pain at the fracture site.

Serious adverse events (SAEs) will also be documented at each visit. X-rays of the participant’s fractured hip are required at enrollment (baseline), post-operative, and at 6 weeks, 3 months, 6 months, 9 months, and 12 months post-surgery (Table 1). As the primary outcome includes radiographic outcomes, it is important that X-rays be obtained at all follow-up visits. Magnetic resonance imaging (MRI) or other advanced imaging studies are not required and may be ordered at the discretion of the attending surgeon. Clinical notes may also be requested. If additional imaging studies are obtained, they will be sent to the Methods Centre for outcome adjudication. In addition, at the 12-month follow-up visit, any re-operations that may be planned for the participant will be documented.

### Participant retention

Once a patient is enrolled in the trial, the clinical site will make every reasonable effort to follow the participant for the entire duration of the study period. The expected follow-up rate for this study is greater than 90% based on similar fracture trials performed by the study investigators [40, 41]. To maximize participant retention, all possible attempts should be made to collect as much data as possible and to reduce loss to follow-up. We have implemented procedures to improve participant retention [42]. Clinical site personnel are responsible for implementing these procedures, as well as developing their own local procedures, to attain this follow-up rate.

Participants may discontinue their participation in the FAITH-2 study at any time. If a participant wishes to withdraw their consent from the study, we will try to reduce the demands of the study and help to retain the study participant by either asking them to return for a clinic visit at the 12-month follow-up only to ensure items in the primary outcome are completed, ask for their permission for research personnel to contact them by telephone to ask about their status, or ask for permission to access their medical chart to identify information about their status.

We will only deem participants lost to follow-up after all exhaustive measures have been taken to locate the participant. Participants should not be deemed lost to follow-up until the 12-month visit is due and all attempts to contact the participant have been exhausted.

### Blinding

Surgeons, research personnel, participants, and the adjudicator cannot be blinded to the treatment allocation of the surgical interventions (SHS versus cancellous screws). The data analyst, the Steering Committee, and those interpreting the study results will be blinded to the surgical treatment allocation.

The complete blinding of the nutritional supplement will be achieved by using vitamin D_3_ and placebo liquid products that are indistinguishable. This will ensure that the surgeon, participants, research personnel, the adjudicator, the data analyst, and the Steering Committee are blinded to the participants’ nutritional supplementation allocation. An unblinding procedure will be made available when necessary.

### Sample size consideration

Feasibility objectives in our pilot study do not lend themselves to traditional quantitative sample size calculations. We plan to conduct the pilot study over a 2-year period, of which 12 months will be dedicated to patient recruitment. We proposed a sample size of 60 patients to assess the feasibility of a definitive large RCT.

### Statistical analysis of outcomes

We will adopt the CONSORT extension to pilot trials in reporting the results of this pilot trial. [43]

### Analysis of feasibility outcomes

We will use descriptive statistics, reported as count and percentage or mean and standard deviation depending on the type of variable to summarize our feasibility outcomes of the initiation of clinical sites (locations and timelines), rate of participant enrolment, rate of protocol adherence (the number of errors in randomization, the number of crossovers between SHS and cancellous screw treatment groups, adherence to the daily vitamin D supplementation), proportion of participants with complete follow-up at 12 months post-fracture, and level of data quality (Table 2).

**Table 2:** Planned analysis of feasibility and clinical outcomes

Variable/outcome	Type of outcome	Hypothesis for surgical treatments	Hypothesis for biologic treatments	Outcome measures	Method of analysis
Initiation of clinical sites	Feasibility	N/A	N/A	Reporting of locations and timelines of initiation	Count and percentage or mean and standard deviation or median and interquartile range
Rate of participant enrolment	Feasibility	N/A	N/A	Number of participants enrolled	Count and percentage
Rate of protocol adherence	Feasibility	N/A	N/A	Number of errors in randomization	Count and percentage
				Number of crossovers between SHS and cancellous screw treatment groups	
				Adherence to the daily vitamin D supplementation	
Proportion of participants with complete follow-up at 12 months post-fracture	Feasibility	N/A	N/A	Number of participants who complete follow-up at 12 months post-fracture	Count and percentage
Level of data quality	Feasibility	N/A	N/A	Completeness of data	Count and percentage
Composite of patient-important outcomes (re-operation, femoral head osteonecrosis, severe femoral neck malunion, nonunion)	Clinical	The risk of patient important outcomes will be lower in the SHS treatment arm compared to cancellous screw treatment arm	The risk of patient important outcomes will be lower in the vitamin D treatment arm than in the placebo arm	Unplanned re-operation related to the treatment of the femoral neck fracture anytime within 12 months	Cox regression
				Evidence of femoral head osteonecrosis on X-rays or MRI	
				Evidence of severe femoral neck malunion on X-rays (shortening of > 10 mm in any plane on follow-up X-rays)	
				Nonunion, defined as the failure of the fracture to progress towards healing defined as a RUSH score below 18 at 6 months or greater post-injury	
Health-related quality of life and patient-reported function	Clinical	Health-related quality of life and patient-reported function will be better in the SHS treatment arm compared to cancellous screw treatment arm	Health-related quality of life and patient-reported function will be better in the vitamin D treatment arm than in the placebo arm	Short Form-12 (SF-12) and Hip Outcome Score (HOS)	*T* test
Fracture healing complications	Clinical	Rates of fracture healing complications will be lower in the SHS treatment arm compared to cancellous screw treatment arm	Rates of fracture healing complications will be lower in the vitamin D treatment arm than in the placebo arm	Evidence of complications reported by patients or evident on X-rays	Cox regression
Radiographic fracture healing	Clinical	Fractures will heal faster in the SHS treatment arm compared to cancellous screw treatment arm	Fractures will heal faster in the vitamin D treatment arm than in the placebo arm	Evidence of fracture healing on X-rays	Cox regression

### Analysis of the clinical outcome

If the feasibility analysis demonstrates a successful pilot study and the study team makes the decision to proceed with a definitive trial and include the participants enrolled in the definitive trial, we will not analyze the clinical outcomes data for the pilot study. Conversely, if the pilot study participants are not going to be included in the definitive trial, we will analyze the clinical outcomes as per below. We will consider the clinical analyses of the pilot study data as exploratory. Therefore, we will not adjust for multiple testing and not draw definitive conclusions [44]. Additionally, we will not conduct the subgroup analyses described below in the analysis of the pilot study data, as per the CONSORT recommendations [43].

The baseline characteristics, fracture characteristics, surgical details, and peri-operative care data will be summarized using descriptive statistics reported as means (standard deviation) or medians (first quartile, third quartile) for continuous variables depending on their distribution and counts (percent) for categorical variables.

The first analysis of clinical outcomes will be a Cox regression with main effects for implant type and supplementation, and the interaction between the two. The analysis will be adjusted for 1) femoral neck fracture displacement (displaced versus undisplaced), 2) presence of an ipsilateral femoral shaft fracture (present versus absent), and 3) geographic region of recruiting center (industrializing countries versus industrialized countries). If the interaction is not significant at alpha = 0.05, we will perform two independent Cox regression analyses between the treatment groups (SHS versus cancellous screws and separately vitamin D supplementation versus placebo) with time to the composite of patient important outcomes as the endpoint (Table 2). Similarly, the analyses will be adjusted for (1) femoral neck fracture displacement (displaced versus undisplaced), 2) presence of an ipsilateral femoral shaft fracture (present versus absent), and 3) geographic region of recruiting center (industrializing countries versus industrialized countries). If the interaction is significant, we will keep this interaction term in all subsequent analyses, and present two hazard ratios for the surgical intervention and two hazard ratios for the nutritional supplementation (i.e., We will report a hazard ratio for SHS versus cancellous screws in those who receive vitamin D, and a separate hazard ratio for SHS versus cancellous screws in those who receive placebo. We will also report a hazard ratio for vitamin D versus placebo in those who receive SHS, and a separate hazard ratio for vitamin D versus placebo in those who receive cancellous screws.)

HRQL, patient-reported function, and fracture healing complications will be summarized using means and 95% confidence intervals, or percentages and counts. Longitudinal models will be used to explore the effect of treatment group and time on the HOS and SF-12 patient-reported outcomes. A Cox regression analysis will be conducted to evaluate radiographic fracture healing (Table 2).

For patients with ipsilateral femoral shaft fractures, a time-to-event analysis will be conducted to assess the time to radiographic fracture healing of the ipsilateral femoral shaft fracture. The method of shaft fracture fixation, fracture-related complications, and re-operations on the ipsilateral femoral shaft fracture will be summarized using counts and percentages.

Missing data will be handled using multiple imputation. All outcome analyses will adhere to the intention-to-treat principle. We will use SPSS Version 25 to perform all analyses.

### Steering committee

The Steering Committee is comprised of orthopedic surgeons, vitamin D experts, a statistician, and research methodologists. The Steering Committee will provide guidance and direction to the trial.

### Data safety and monitoring committee

The Data Safety and Monitoring Committee (DSMC) is comprised of three members who remain completely independent of the study investigators. The DSMC members include a biostatistician (Chair) and two orthopedic surgeons with prior trial experience. The DSMC will review accumulated safety data (i.e., SAEs and fracture-related adverse events) from the trial and advise the Principal Investigators and the Steering Committee on items related to participant safety.

### Adjudication

The adjudicator for the definitive trial will be an orthopedic surgeon with clinical trial experience who treats high energy femoral neck fracture patients. The outcomes that the adjudicator will independently assess include:

1. Situations where eligibility is in doubt.
2. Radiographic characteristics and quality of the surgery.
3. Patient important outcomes that define the primary outcome (re-operation, femoral head osteonecrosis, severe femoral neck malunion, or nonunion (using the RUSH score))
4. Fracture healing complications
5. Radiographic fracture healing

All clinical sites will submit X-rays and any additional imaging studies (such as a hip MRI or other advanced imaging studies) to be included in the adjudication process. Clinical notes may also be requested. Additional information will be requested from the clinical site to clarify areas of uncertainty.

## Discussion

If the pilot study demonstrates feasibility and minimal changes to the protocol are needed, we will transition directly into the definitive trial and the pilot study participants will be included in the definitive trial. If the feasibility analysis shows that the trial as designed is not feasible and major changes are required to the protocol, then the pilot study participants will not be included in the definitive trial. We have used the above approach in our prior orthopedic trials [40, 45–48]. The methodology of the planned definitive trial is described in detail below.

### Primary and secondary clinical research questions for the planned definitive trial

The same primary and secondary clinical research questions for the pilot study, as described above, will be used for the planned definitive trial.

### Subgroup research questions for the planned definitive trial

1. In patients with femoral neck fractures, will the magnitude of treatment effect favoring the SHS treatment arm be higher in displaced fractures compared to undisplaced fractures 12-months post-injury?
2. In patients with femoral neck fractures, will the magnitude of treatment effect favoring the SHS treatment arm be higher in fractures treated emergently versus non-emergently 12-months post-injury?
3. In patients with femoral neck fractures, will the magnitude of treatment effect favoring the vitamin D treatment arm be higher in displaced fractures compared to undisplaced fractures 12-months post-injury?

### Outcome measure for the planned definitive trial

The same primary and secondary clinical outcomes for the pilot study, as described above, will be used for the planned definitive trial.

### Analysis of the clinical outcome for the planned definitive trial

The same analysis of the clinical outcome for the pilot study, as described above, will be performed for the planned definitive trial, as well as two additional subgroup analyses. Two a priori subgroup analyses are planned using logistic regression models for the primary clinical outcome: 1) the magnitude of treatment effect of the surgical and nutritional supplementation interventions, independently, on undisplaced versus displaced femoral neck fractures and 2) the magnitude of treatment effect of the surgical intervention on emergent (< 8 h) versus non-emergent (≥ 8 h) internal fixation.

### Sample size consideration for the planned definitive trial

The choice of sample size for our definitive trial is based upon independent comparisons of the SHS versus multiple cancellous screw and vitamin D supplementation versus placebo for the primary outcome (patient important outcomes). This is based on the assumption that both interventions will act independently. We will use an alpha level of 0.05 for the primary outcome and all statistical hypotheses will be two-sided.

The preliminary sample size calculations are based on the limited published literature. A pooled estimate from 18 published studies revealed a combined femoral head osteonecrosis and fracture nonunion incidence of 25% [49]. In addition to femoral head osteonecrosis, the composite primary outcome includes other events such as re-operation and severe femoral neck malunion. These additional complications are expected to increase the composite event rate to approximately 40%; therefore, a conservative event rate of 30% is assumed in the cancellous screw and placebo groups.

The trial is powered for a relative risk reduction (RRR) of 33% for each of the treatment comparisons: SHS versus multiple cancellous screws and vitamin D supplementation versus placebo. This RRR estimate is based on the best available literature and coincides with a 10% absolute risk reduction that was deemed to be clinically significant by over 500 surveyed surgeons [21]. For the surgical comparison, Gardner et al. and Chen et al. reported an 86% and 100% RRR for re-operation using a SHS [18, 50]. Other more heterogeneous retrospective studies by Liporace et al. and Razik et al. suggest RRRs from SHS between 50 and 88% for osteonecrosis and 63% for nonunion [19, 20]. Since these are uncontrolled retrospective studies, a conservative effect size estimate is maintained. With regard to the expected treatment effect of vitamin D supplementation, there are no clinical studies that quantify its efficacy to reduce fracture healing complications. Despite the lack of direct data, vitamin D supplementation following acute fractures has demonstrated a 40% increase in fracture callus density in human participants and an 80% increase in mechanical strength in animal model [11, 12]. Experimental evidence also suggests benefits of increased fracture area vascularity and improved healing that may also contribute to the treatment effect of vitamin D supplementation. We have also assumed the same RRR of 33% in the vitamin D treatment arm. Considering a 30% event rate in the cancellous screw and placebo group, an RRR of 33% will give a 20% event rate in the SHS and placebo group. Therefore, the event rate for the total placebo group (combined over cancellous screw and SHS groups) is expected to be 25%. Similarly, an RRR of 33% due to vitamin D supplementation will result in a 20% event rate in the cancellous screw and vitamin D supplementation group. This leads to an expected event rate of 25% for the total cancellous screw group (combined over both the placebo and vitamin D supplementation groups). Therefore, our sample size calculation is based on a control group event rate of 25% and an RRR of 33%.

Therefore, for the definitive trial, we will recruit a sample size of 808 patients with full follow-up. Based on an anticipated 10% loss to follow-up, 898 patients will need to be enrolled in the FAITH-2 trial [51].

### Dissemination

Study results for the pilot stud and the definitive study will be disseminated through a publication in an academic journal and through presentations at relevant orthopedic conferences. Every attempt will be made to minimize the amount of time between the completion of data collection and the release of study findings.

### Potential impact of study

The results of this pilot study will inform the feasibility and design of the definitive trial. If the pilot study is successful, the definitive FAITH-2 trial may have the potential to provide evidence to improve the management of young femoral neck fractures. High-quality evidence is currently needed as femoral neck fractures in patients 18–60 years of age are a major source of disability worldwide and there exist conflicting evidence and a lack of surgeon consensus in treatments for this type of injury. More broadly, the definitive FAITH-2 trial will contribute toward expanding a network of collaboration from which we expect further surgical trials to follow. This multi-center international collaboration has the rigorous study design, organization, and execution standards necessary to provide an adequately powered, high-quality trial to guide treatment decisions.

## Additional file

Additional file 1: SPIRIT 2013 Checklist. (DOC 121 kb)

## Abbreviations

25(OH)D: 25-Hydroxyvitamin D
ADL: Activities of daily living
AVN: Avascular necrosis
DSMC: Data Safety and Monitoring Committee
FAITH-2: Fixation using Alternative Implants for the Treatment of Hip Fractures
HIREB: Hamilton Integrated Research Ethics Board
HOS: Hip Outcome Score
HRQL: Health-Related Quality of Life
IRB: Institutional Review Board
IU: International units
MRI: Magnetic resonance imaging
RCT: Randomized controlled trial
RRR: Relative risk reduction
REB: Research Ethics Board
RUSH: Radiographic Union Score for Hip
SAE: Serious adverse event
SF-12: Short Form-12
SHS: Sliding hip screw
